# Birth characteristics and the risk of childhood rhabdomyosarcoma based on histological subtype

**DOI:** 10.1038/sj.bjc.6605484

**Published:** 2009-12-08

**Authors:** S Ognjanovic, S E Carozza, E J Chow, E E Fox, S Horel, C C McLaughlin, B A Mueller, S Puumala, P Reynolds, J Von Behren, L Spector

**Affiliations:** 1 Division of Pediatric Epidemiology and Clinical Research, Department of Pediatrics, University of Minnesota, Minneapolis, MN, USA; 2 Masonic Cancer Center, University of Minnesota, Minneapolis, MN, USA; 3 Department of Public Health, Oregon State University, Corvallis OR, USA; 4 Fred Hutchinson Cancer Research Center, Seattle, WA, USA; 5 Cancer Epidemiology and Surveillance Branch, Austin, TX, USA; 6 Department of State Health Services, Austin, TX, USA; 7 New York State Cancer Registry, New York Department of Health, Albany, NY, USA; 8 Northern California Cancer Center, Berkeley, CA, USA

**Keywords:** birth weight, gestational age, paediatric rhabdomyosarcoma

## Abstract

**Background:**

Little is known about risk factors for childhood rhabdomyosarcoma (RMS) and the histology-specific details are rare.

**Methods:**

Case–control studies formed by linking cancer and birth registries of California, Minnesota, New York, Texas and Washington, which included 583 RMS cases (363 embryonal and 85 alveolar RMS) and 57 966 randomly selected control subjects, were analysed using logistic regression. The associations of RMS (overall, and based on embryonal or alveolar histology) with birth weight across five 500 g categories (from 2000 to 4500 g) were examined using normal birth weight (2500–3999 g) as a reference. Large (>90th percentile) and small (<10th percentile) size for gestational age were calculated based on birth weight distributions in controls and were similarly examined.

**Results:**

High birth weight increased the risk of embryonal RMS and RMS overall. Each 500 g increase in birth weight increased the risk of embryonal RMS (odds ratio (OR)=1.27, 95% confidence interval (CI)=1.14–1.42) and RMS overall (OR=1.18, 95% CI=1.09–1.29). Large size for gestational age also significantly increased the risk of embryonal RMS (OR=1.42, 95% CI=1.03–1.96).

**Conclusions:**

These data suggest a positive association between accelerated *in utero* growth and embryonal RMS, but not alveolar RMS. These results warrant cautious interpretation owing to the small number of alveolar RMS cases.

Rhabdomyosarcoma (RMS) is the commonest soft-tissue sarcoma in children below 15 years, with half the cases occurring before the age of 10 years (Ries, 1999). Owing to the early age of onset, *in utero* exposures have been suggested to have a role in its aetiology, including *in utero* X-ray exposure (Grufferman, 1991, 2009), maternal drug use (Grufferman *et al*, 1993, 1982), advanced maternal age(Grufferman, 1991) and stillbirths (Ghali *et al*, 1992). In addition, several familial syndromes (Diller *et al*, 1995; DeBaun and Tucker, 1998; Samuel *et al*, 1999; Tekin *et al*, 2002; Meyer *et al*, 2004; Aoki *et al*, 2005; Gripp, 2005; Ferrari *et al*, 2007; Moschovi *et al*, 2007; Trahair *et al*, 2007; Li and Fraumeni, 1969a, 1969b) and a larger prevalence of congenital malformations (Ruymann *et al*, 1988) among affected children suggest that genetic predisposition may be important. However, collectively these factors account for only a small fraction of cases.

High birth weight has been associated with several childhood malignancies, and recent evidence suggests that accelerated intrauterine growth may increase RMS risk, although based on only 27 cases (Laurvick *et al*, 2008). Further, the two major histological subtypes (embryonal and alveolar RMS) have distinct localisations, ages of onset, incidence and survival trends (Ries, 1999; Ognjanovic *et al*, 2009) and distinct tumour gene expression signatures (Davicioni *et al*, 2006; Wachtel *et al*, 2006; Lae *et al*, 2007), suggesting that their aetiologies may be distinct. Embryonal RMS is characterised by earlier age of onset than alveolar RMS; the majority of embryonal RMS cases are diagnosed in the first decade of life, with more than half of cases occurring before the age of 5 years. As an equal number of alveolar RMS is diagnosed in the first and second decades of life (Ognjanovic *et al*, 2009), we hypothesised that factors affecting *in utero* growth, resulting in high birth weight would be more important in embryonal than in alveolar RMS.

To assess the role of birth characteristics and the risk of RMS and its two major subtypes, we used pooled data from five states (Minnesota, California, New York (excluding New York City), Washington and Texas) with case–control data sets by having linked cancer and birth registries (Spector *et al*, 2009).

## Materials and methods

Institutional review board approvals were obtained from each participating institution and State department of health. The selection of cases and controls varied by state and has been previously described in detail (Puumala *et al*, 2009). The age range was 28 days to 14 years, except in California, wherein only cases up to age 4 years were available. The contribution of cases and controls by each state to this pooled analysis is shown in detail in Table 1. Controls were matched to cases in all states on birth year, whereas additional matching on sex was performed in California and Texas (Table 1). Controls were frequency matched, except in California, wherein individual matching was used. The number of controls per case varied by state and ranged from 1 to 10 controls per case. Children with documented Down's syndrome in their birth records were excluded from analyses (*n*=100), but possibly not completely, because such reporting was not implemented in Texas before 1984 and in Washington before 1989.

The information available for cases included RMS diagnoses and the age of onset. Cases were classified according to the International Classification of Childhood Cancer (ICCC-3), third edition (Steliarova-Foucher *et al*, 2005) using the following histology codes: 8910/3, embryonal RMS; 8991/3, alveolar RMS; 8900/3, not otherwise specified; 8901/3, pleomorphic; 8902/3, mixed type; and 8912/3, spindle cell. In our analyses, RMS overall included all six categories, whereas embryonal and alveolar RMS were also analysed separately.

The perinatal characteristics available from birth records were birth weight, gestational age, sex, birth order, plurality (singleton or multiple birth), year of birth, parental age and race/ethnicity and maternal years of education. Gestational age clinical estimate was provided by each state, except California, which provided only the date of last menstrual period (LMP) that was used for gestational age calculation. Some states also provided the LMP information; therefore, a combined gestational age variable was developed that gave preference to the calculated estimate when available and used clinical estimate otherwise. Gestational ages less than 20 or more than 45 weeks were deemed implausible, as were birth weights less than 350 g, and were excluded from analyses. Maternal education was available from all states only after 1992 and was, therefore, included in secondary analysis. Using birth records data, we calculated size for gestational age based on the distribution observed in controls (described below). Separate distributions were used for males and females.

Birth weight categories were selected to distinguish very low birth weight (<2000 g), low birth weight (2000–2499 g), normal weight (2500–3999 g), high birth weight (4000–4499 g) and very high birth weight (>4500 g). The very low birth weight category was assigned higher than the standard cutoff at <1500 g, because only seven cases weighed less than 2000 g. Normal birth weight was used as a reference group for comparisons. Gestational age was categorised as 20–36 (preterm), 37–40 (term) and >40 weeks (post-term). Size for gestational age was analysed using an internal standard (the distribution in controls). The tenth and ninetieth percentiles were used as cut points for small for gestational age and large for gestational age (LGA), respectively. We used this internal standard rather than other published external standards because of the large number of controls available and the long span of the study. For plurality, we distinguished single *vs* multiple births. Birth order was categorised as first, second, third, fourth or higher order. Parental age categories consisted of <20, 20–24, 25–29, 30–34, and >35 years. Maternal education categories consisted of <12, 12, 13–16, >17 years.

### Statistical analysis

All analyses were carried out using SAS version 9.1 (Cary, NC, USA). Unconditional logistic regression was used to calculate adjusted odds ratios (OR) and 95% confidence intervals (CI) to examine the associations between perinatal characteristics and RMS and its two major subtypes. Individual matching in California was broken for the purpose of pooled analysis. Results of analyses in subgroups which had fewer than four (RMS overall) or two cases (for RMS subtypes) were not reported. Risk estimates were adjusted for available covariates. After adjustment for state/study centre, the choice of other variables evaluated as possible confounders included those known or suspected to be associated with inappropriate fetal growth (Das and Sysyn, 2004; Valero De Bernabe *et al*, 2004).

## Results

This study included a total of 583 RMS cases; of these, 363 were embryonal and 85 alveolar RMS. The sex distribution varied by subtype, with embryonal RMS affecting 30% more males than females (Table 2). As this subtype comprised 62% of all RMS cases, a similar proportion was observed for the entire group. The proportion of mothers and fathers older than 35 years at child's birth was greater in cases than controls (data not shown). However, mean age of mothers of RMS patients was similar to controls (27.2 and 26.7 years, respectively), as were those of fathers (30.2 and 29.7 years, respectively). Maternal and paternal ages were highly correlated (Pearson's correlation coefficient=0.74). As anticipated, the alveolar RMS cases were older on average, with 53% diagnosed at <4 years compared with 68% of embryonal RMS cases. A similar proportion of cases and controls were born from singleton or multiple pregnancies. The proportion of ‘other’ mothers (that is, not white, black or Asian race) was significantly higher for embryonal RMS cases than controls.

Table 2 shows the associations between birth characteristics of the child and RMS risk. The multivariate models included birth year, state, maternal age, race, child's sex, gestational age, plurality, birth order and birth weight, and were adjusted for all other covariates and matching factors. For parental characteristics, we observed the previously reported (Grufferman *et al*, 1982) association between maternal age over 35 years and increased risk of RMS (OR=1.35, 95% CI=1.01–1.81), suggesting that embryonal histology was driving this association (OR=1.54, 95%CI=1.08–2.19). A 1-year increase in maternal age was associated with 3 and 4% increases in RMS overall and embryonal RMS risk, respectively. Paternal age over 35 years was not significantly associated with increased RMS risk; however, a 1-year increase in paternal age lead to 2% increased risk of RMS overall and embryonal RMS. For maternal race, a significant association between embryonal RMS and the category of ‘other’ race, reflecting non-white, non-black or non-Asian population, such as Hispanic or American Indians, was observed. As this is a very heterogeneous category with only 11 embryonal RMS cases, it probably represents a chance finding.

Higher birth order was inversely associated with risk of RMS overall and embryonal RMS, but not alveolar RMS. Compared with the first-born child, RMS point estimate for second-born child was OR=0.82 (95% CI=0.67–1.00) and for third-born child it was OR=0.70 (95%CI=0.54–0.91). This reduced risk reflects the significant associations with birth order observed for embryonal RMS, whereas no associations were observed for alveolar RMS (Table 2).

Higher birth weight, however, was associated with increased risk of RMS overall and embryonal RMS, with OR=1.19 (95% CI=1.09–1.29) and OR=1.27 (95% CI=1.14–1.42) per 500 g increase in birth weight, respectively. Despite the trend, none of the individual categories showed a significant association with these risks. Only two alveolar RMS cases had birth weights below normal (<2500 g); hence, we could only compare cases with normal birth weight (<4000 g) (reference) with those with high birth weight (4000–4500 g), and we observed no association. We observed no association with gestational age for RMS or either of its subtypes. The majority of cases born preterm were between 32 and 36 weeks gestation, and only two cases were born before 32 weeks.

To distinguish the effect of birth weight *per se* from that of accelerated fetal growth, we calculated size for gestational age as a surrogate measure for accelerated fetal growth (Table 3). Being LGA was associated with increased risk of RMS that reached statistical significance for embryonal RMS (OR=1.43, 95% CI=1.03–1.96) only.

Given the different control–case ratios in participating states, sensitivity analyses were also conducted to determine whether the number of controls per case affected the observed ORs. Limiting the control number to one per case did not appreciably affect the results in the main analysis (data not shown).

## Discussion

Children whose birth weight was high had an increased risk of RMS overall and of embryonal RMS in particular. Trend analysis showed that the birth weight increase per 500 g increment increased the odds of RMS and its embryonal subtype. The examination of size for gestational age showed that LGA babies had increased risk of developing embryonal RMS, whereas no associations between birth weight alone or birth weight for gestational age were observed for alveolar RMS. Our results support the hypothesis that the two major RMS subtypes, embryonal and alveolar RMS, have different aetiologies. The associations with RMS overall primarily reflected the associations observed for embryonal RMS, as the majority of cases belonged to this subtype.

This is the first study to show that accelerated *in utero* growth, measured by higher than expected weight for gestational age, increases the risk of embryonal RMS. The only previous study of relevance suggested an increased risk with increasing birth weight and intrauterine growth (Laurvick *et al*, 2008), but the small sample size precluded precise estimates. We and others have not only shown that high birth weight increases the risk of RMS thereby extending the associations reported for childhood cancers, including leukaemia (Caughey and Michels, 2009), brain tumours (Harder *et al*, 2008) and Wilms tumour (Schuz *et al*, 2001), but also directly linked it to the extent of growth *in utero*. Our study supports the view that size for gestational age provides insight beyond that of birth weight *per se* (Laurvick *et al*, 2008) and can identify new high-risk groups (Schuz *et al*, 2001). We observed no association between gestational age and RMS risk, which may also reflect the fact that only 26 embryonal and 7 alveolar RMS cases were born prematurely, spread across 5 weeks before term (32–36 weeks), whereas only two RMS cases were born very prematurely (more than 5 weeks).

Temporal trends in fetal growth show that the birth weight of term infants has increased over the last two decades of the previous century (Kramer *et al*, 2002), attributed primarily to rising maternal pre-pregnancy BMI, greater gestational weight gain and more prevalent gestational diabetes. These maternal anthropometric factors affect maternal basal metabolic rates and increase insulin-like growth factor-1 (IGF-1) levels, which lead to larger fetal size (Chellakooty *et al*, 2004; Lof *et al*, 2005). Insulin-like growth factors have a major role in fetal growth and development, whereas their mitotic and anti-apoptotic properties can also enhance tumour growth and may underlie the association between larger fetal size and increased childhood cancer risk (Greaves, 2002). Indeed, an aetiological role of IGFs has been proposed for embryonal RMS, in which chromosomal deletions, which frequently affect the area of imprinted genes, including IGF-2, lead to aberrant IGF-2 expression (Scrable *et al*, 1989; Rainier *et al*, 1993). Beckwith–Wiedemann syndrome is associated with fetal overgrowth resulting from loss of imprinting in the IGF-2-containing region on chromosome 11, and this syndrome increases RMS risk (Dagher and Helman, 1999). Therefore, the differences between embryonal and alveolar RMS risk associated with fetal growth may partly reflect differences in underlying genetic predispositions likely to be reflected in different growth patterns.

We also found that RMS cases were more likely to be first born, as increasing birth order was associated with progressively decreasing RMS risk owing to the embryonal subtype; no association was observed for alveolar RMS. The similar findings observed in childhood leukaemia prompted the suggestion that later exposures to infections, an established risk factor for leukaemia, may have been more frequent among the first-born children (Schuz *et al*, 2001). Although no direct associations between infections and RMS have been reported, maternal or child's antibiotic use was associated with increased risk of RMS (Grufferman *et al*, 1982; Hartley *et al*, 1988), something we were not able to evaluate. However, whether these associations were confined to embryonal RMS and to first-born children only was not examined.

We confirmed previously reported associations with increasing maternal age, and have shown that this is significant for embryonal histology only. In addition, we have observed that each 1-year increase in paternal age is associated with a slight, but significant, increase in RMS overall and embryonal RMS risk. Maternal education was not associated with RMS risk, overall or by subtype; however, this may be due to the limited availability of this variable.

The main limitations of this study include multiple comparisons and potential measurement error in the birth certificate data. Although maternal age, birth weight, birth order and plurality have been shown to be accurate in validation studies (Northam and Knapp, 2006), gestational age reporting is likely to be less reliable on birth certificates. Moreover, this was assessed by two different methods depending on the state, which increased the complexity of analysis and potentially introduced further error, though we have no reason to suspect that this would have been differential. Multivariate analyses were adjusted for potential confounders. However, owing to categorisation of the variables, some residual confounding may have remained. Strengths include that this was population based and, therefore, avoided referral-type biases from single-center studies or from those relying on subjects only identified in clinical trials. In addition, the ascertainment of exposures preceded diagnosis, thereby avoiding any reporting bias by case–control status.

Our study further supports the view that embryonal RMS may be associated with growth characteristics, suggesting that accelerated *in utero* growth may have an important role. However, caution is warranted in interpreting the results related to alveolar and other RMS histology subtypes, given their small numbers, and further studies are needed.

## Figures and Tables

**Table 1:** Details of information provided by each state in the pooled analysis

**State**	**Ages at diagnosis**	**Years of diagnosis**	**Years of birth**	**RMS cases**	**No. of controls**	**Matching factors**
Minnesota	28 days to 14 years	1988–2004	1976–2004	73	8735	Birth year
Washington	28 days to 14 years	1980–2004	1980–2004	74	23 728	Birth year, sex
California	28 days to 4 years	1988–1997	1983–1997	136	8730	Birth year, sex
New York	28 days to 14 years	1985–2001	1970–2001	152	12 041	Birth year
Texas	28 days to 14 years	1990–1998	1975–1998	148	4732	Birth year

Abbreviation: RMS=rhabdomyosarcoma.

**Table 2:** Perinatal characteristics, including birth weight and the risk of childhood RMS, and its two major subtypes (embryonal RMS and alveolar RMS)

	**All RMS**		**Embryonal RMS**		**Alveolar RMS**	
**Variable**	**OR^a^ (*N*)**	**95% CI**	**OR^a^ (*N*)**	**95% CI**	**OR^a^ (*N*)**	**95% CI**
*Maternal race*						
White	Ref (494)		Ref (312)		Ref (69)	
Black	0.93 (43)	0.67–1.29	0.76 (22)	0.48–1.21	1.43 (9)	0.70–2.92
Asian	1.01 (26)	0.66–1.54	1.02 (18)	0.61–1.71	(3)	
Other	1.44 (14)	0.83–2.51	**2.04** (11)	**1.09**–**3.84**	(2)	
						
*Maternal age (years)*						
<20	0.84 (65)	0.62–1.15	0.70 (35)	0.46–1.05	1.08 (11)	0.50–2.33
20–24	0.81 (131)	0.64–1.02	0.78 (79)	0.58–1.05	0.91 (21)	0.51–1.64
25–29	Ref (188)		Ref (118)		Ref (28)	
30–34	1.06 (131)	0.84–1.33	1.05 (83)	0.78–1.41	0.77 (15)	0.40–1.48
⩾35	**1.35** (68)	**1.01**–**1.81**	**1.54** (48)	**1.08**–**2.19**	1.27 (10)	0.60–2.69
Per 1-year increase	**1.03**	**1.01**–**1.04**	**1.04**	**1.02**–**1.06**	1.01	0.97–1.05
						
*Paternal age*^b^ *(years)*						
<20	0.84 (17)	0.49–1.44	0.50 (7)	0.22–1.15	2.57 (5)	0.83–7.98
20–24	0.75(74)	0.56–1.00	0.72 (46)	0.50–1.04	1.26 (11)	0.58–2.76
25–29	Ref (158)		Ref (102)		Ref (17)	
30–34	1.04 (139)	0.82–1.32	1.01 (86)	0.75–1.36	1.70 (23)	0.88–3.27
⩾35	1.21 (119)	0.94–1.56	1.24 (77)	0.90–1.69	1.82 (18)	0.90–3.68
Per 1-year increase	**1.02**	**1.01**–**1.03**	**1.02**	**1.00**–**1.04**	1.02	0.98–1.06
						
*Child's sex*						
Male	**1.27** (348)	**1.07**–**1.51**	**1.29** (221)	**1.04**–**1.60**	1.19 (47)	0.77–1.85
Female	Ref (235)		Ref (142)		Ref (38)	
						
*Gestational age*						
⩽36 weeks	1.02 (42)	0.71–1.45	1.02 (26)	0.65–1.60	0.92 (7)	0.42–2.04
37–40 weeks	Ref (349)		Ref (218)		Ref (52)	
>40 weeks	0.98 (173)	0.81–1.18	0.94 (105)	0.74–1.19	0.93 (24)	0.56–1.53
						
*Plurality*						
Single birth	Ref (570)		Ref (356)		(82)	
Multiple birth	1.32 (13)	0.74–2.36	1.13 (7)	0.52–2.48	(3)	
						
*Birth order*						
First	Ref (261)		Ref (170)		Ref (34)	
Second	0.82 (178)	0.67–1.00	**0.72** (105)	**0.55**–**0.92**	1.01 (25)	0.59–1.74
Third	**0.70** (82)	**0.54**–**0.91**	**0.57** (48)	**0.40**–**0.80**	1.23 (15)	0.64–2.34
Fourth +	0.75 (59)	0.55–1.02	0.69 (38)	0.47–1.01	1.12 (11)	0.51–2.48
						
*Birth weight*						
<2000 g	0.52 (7)	0.22–1.24	0.59 (4)	0.20–1.71	(1)	
2000–2499 g	0.66 (15)	0.38–1.14	0.67 (9)	0.33–1.35	(1)	
2500–3999 g	Ref (476)		Ref (294)		Ref^c^ (75)	
4000–4499 g	1.08 (64)	0.82–1.42	1.21 (44)	0.87–1.69	0.48 (5)	0.19–1.19
⩾4500 g	1.21 (15)	0.70–2.08	1.55 (11)	0.84–2.86	(2)	
Per 500 g increase	**1.18**	**1.09**–**1.29**	**1.27**	**1.14**–**1.42**	0.92	0.74–1.14

Abbreviations: CI=confidence interval; OR=odds ratio; RMS=rhabdomyosarcoma.

a Adjusted for birth year, state, maternal age, maternal race, child's sex, plurality and birth order.

b Adjusted for birth year, state, maternal race, child's sex, plurality, gestational age, birth weight and birth order.

c Birth weight <4000 g was used as a reference and compared with birth weight ⩾4000 g. Terms given in bold mark statistically significant results.

**Table 3:** Associations between size for gestational age and RMS risk

	**All RMS**		**Embryonal RMS**		**Alveolar RMS**	
**Variable**	**OR**	**95% CI**	**OR**	**95% CI**	**OR**	**95% CI**
*Size for gestational age* a						
Small	0.91	0.68–1.23	0.91	0.62–1.33	0.94	0.45–1.96
Average	Ref		Ref		Ref	
Large	1.22	0.93–1.61	**1.42**	**1.03**–**1.96**	0.93	0.42–2.03

Abbreviations: CI=confidence interval; OR=odds ratio; RMS=rhabdomyosarcoma.

a Adjusted for birth year, state, maternal age, maternal race, child's sex, plurality and birth order. Terms given in bold mark a statistically significant result.

## References

[bib1] Aoki Y, Niihori T, Kawame H, Kurosawa K, Ohashi H, Tanaka Y, Filocamo M, Kato K, Suzuki Y, Kure S, Matsubara Y (2005) Germline mutations in HRAS proto-oncogene cause Costello syndrome. Nat Genet 37: 1038–1040 16170316 10.1038/ng1641

[bib2] Caughey RW, Michels KB (2009) Birth weight and childhood leukemia: a meta-analysis and review of the current evidence. Int J Cancer 124: 2658–2670 19173295 10.1002/ijc.24225

[bib3] Chellakooty M, Vangsgaard K, Larsen T, Scheike T, Falck-Larsen J, Legarth J, Andersson AM, Main KM, Skakkebaek NE, Juul A (2004) A longitudinal study of intrauterine growth and the placental growth hormone (GH)-insulin-like growth factor I axis in maternal circulation: association between placental GH and fetal growth. J Clin Endocrinol Metab 89: 384–391 14715876 10.1210/jc.2003-030282

[bib4] Dagher R, Helman L (1999) Rhabdomyosarcoma: an overview. Oncologist 4: 34–44 10337369

[bib5] Das UG, Sysyn GD (2004) Abnormal fetal growth: intrauterine growth retardation, small for gestational age, large for gestational age. Pediatr Clin North Am 51: 639–654, viii 15157589 10.1016/j.pcl.2004.01.004

[bib6] Davicioni E, Finckenstein FG, Shahbazian V, Buckley JD, Triche TJ, Anderson MJ (2006) Identification of a PAX-FKHR gene expression signature that defines molecular classes and determines the prognosis of alveolar rhabdomyosarcomas. Cancer Res 66: 6936–6946 16849537 10.1158/0008-5472.CAN-05-4578

[bib7] DeBaun MR, Tucker MA (1998) Risk of cancer during the first four years of life in children from The Beckwith-Wiedemann Syndrome Registry. J Pediatr 132: 398–400 9544889 10.1016/s0022-3476(98)70008-3

[bib8] Diller L, Sexsmith E, Gottlieb A, Li FP, Malkin D (1995) Germline p53 mutations are frequently detected in young children with rhabdomyosarcoma. J Clin Invest 95: 1606–1611 7706467 10.1172/JCI117834 PMC295658

[bib9] Ferrari A, Bisogno G, Macaluso A, Casanova M, D’Angelo P, Pierani P, Zanetti I, Alaggio R, Cecchetto G, Carli M (2007) Soft-tissue sarcomas in children and adolescents with neurofibromatosis type 1. Cancer 109: 1406–1412 17330850 10.1002/cncr.22533

[bib10] Ghali MH, Yoo KY, Flannery JT, Dubrow R (1992) Association between childhood rhabdomyosarcoma and maternal history of stillbirths. Int J Cancer 50: 365–368 1735603 10.1002/ijc.2910500306

[bib11] Greaves M (2002) Childhood leukaemia. BMJ 324: 283–287 11823363 10.1136/bmj.324.7332.283 PMC1122200

[bib12] Gripp KW (2005) Tumor predisposition in Costello syndrome. Am J Med Genet C Semin Med Genet 137C: 72–77 16010679 10.1002/ajmg.c.30065

[bib13] Grufferman S, Gula M, Olshan AF, Falletta JM, Pendergrass TW, Buckley J, Maurer HM (1991) *In utero* x-ray exposure and risk of childhood rhabdomyosarcoma. Paediatr Perinat Epidemiol 5: A6

[bib14] Grufferman S, Ruymann F, Ognjanovic S, Erhardt EB, Maurer HM (2009) Prenatal X-ray exposure and rhabdomyosarcoma in children: a report from the children's oncology group. Cancer Epidemiol Biomarkers Prev 18: 1271–1276 19293315 10.1158/1055-9965.EPI-08-0775 PMC2773469

[bib15] Grufferman S, Schwartz AG, Ruymann FB, Maurer HM (1993) Parents’ use of cocaine and marijuana and increased risk of rhabdomyosarcoma in their children. Cancer Causes Control 4: 217–224 8318638 10.1007/BF00051316

[bib16] Grufferman S, Wang HH, DeLong ER, Kimm SY, Delzell ES, Falletta JM (1982) Environmental factors in the etiology of rhabdomyosarcoma in childhood. J Natl Cancer Inst 68: 107–113 6948120

[bib17] Harder T, Plagemann A, Harder A (2008) Birth weight and subsequent risk of childhood primary brain tumors: a meta-analysis. Am J Epidemiol 168: 366–373 18579539 10.1093/aje/kwn144

[bib18] Hartley AL, Birch JM, McKinney PA, Teare MD, Blair V, Carrette J, Mann JR, Draper GJ, Stiller CA, Johnston HE, Cartwright RA, Waterhouse JAH (1988) The Inter-Regional Epidemiological Study of Childhood Cancer (IRESCC): case control study of children with bone and soft tissue sarcomas. Br J Cancer 58: 838–842 3224086 10.1038/bjc.1988.321 PMC2246858

[bib19] Kramer MS, Morin I, Yang H, Platt RW, Usher R, McNamara H, Joseph KS, Wen SW (2002) Why are babies getting bigger? Temporal trends in fetal growth and its determinants. J Pediatr 141: 538–542 12378194 10.1067/mpd.2002.128029

[bib20] Lae M, Ahn EH, Mercado GE, Chuai S, Edgar M, Pawel BR, Olshen A, Barr FG, Ladanyi M (2007) Global gene expression profiling of PAX-FKHR fusion-positive alveolar and PAX-FKHR fusion-negative embryonal rhabdomyosarcomas. J Pathol 212: 143–151 17471488 10.1002/path.2170

[bib21] Laurvick CL, Milne E, Blair E, de Klerk N, Charles AK, Bower C (2008) Fetal growth and the risk of childhood non-CNS solid tumours in Western Australia. Br J Cancer 99: 179–181 18560404 10.1038/sj.bjc.6604424 PMC2453023

[bib22] Li FP, Fraumeni Jr JF (1969a) Rhabdomyosarcoma in children: epidemiologic study and identification of a familial cancer syndrome. J Natl Cancer Inst 43: 1365–1373 5396222

[bib23] Li FP, Fraumeni Jr JF (1969b) Soft-tissue sarcomas, breast cancer, and other neoplasms. A familial syndrome? Ann Intern Med 71: 747–752 5360287 10.7326/0003-4819-71-4-747

[bib24] Lof M, Olausson H, Bostrom K, Janerot-Sjoberg B, Sohlstrom A, Forsum E (2005) Changes in basal metabolic rate during pregnancy in relation to changes in body weight and composition, cardiac output, insulin-like growth factor I, and thyroid hormones and in relation to fetal growth. Am J Clin Nutr 81: 678–685 15755839 10.1093/ajcn/81.3.678

[bib25] Meyer S, Kingston H, Taylor AM, Byrd PJ, Last JI, Brennan BM, Trueman S, Kelsey A, Taylor GM, Eden OB (2004) Rhabdomyosarcoma in Nijmegen breakage syndrome: strong association with perianal primary site. Cancer Genet Cytogenet 154: 169–174 15474156 10.1016/j.cancergencyto.2004.02.022

[bib26] Moschovi M, Touliatou V, Papadopoulou A, Mayakou MA, Nikolaidou-Karpathiou P, Kitsiou-Tzeli S (2007) Rhabdomyosarcoma in a patient with Noonan syndrome phenotype and review of the literature. J Pediatr Hematol Oncol 29: 341–344 17483716 10.1097/MPH.0b013e31805d8f57

[bib27] Northam S, Knapp TR (2006) The reliability and validity of birth certificates. J Obstet Gynecol Neonatal Nurs 35: 3–12 10.1111/j.1552-6909.2006.00016.x 16466348

[bib28] Ognjanovic S, Linabery AM, Charbonneau B, Ross JA (2009) Trends in childhood rhabdomyosarcoma incidence and survival in the United States, 1975-2005. Cancer 115: 4218–4226 19536876 10.1002/cncr.24465 PMC2953716

[bib29] Puumala SE, Carozza SE, Chow EJ, Fox EE, Horel S, Johnson KJ, McLaughlin C, Mueller BA, Reynolds P, Von Behren J, Spector LG (2009) Childhood cancer among twins and higher order multiples. Cancer Epidemiol Biomarkers Prev 18: 162–168 19124494 10.1158/1055-9965.EPI-08-0660 PMC2705199

[bib30] Rainier S, Johnson LA, Dobry CJ, Ping AJ, Grundy PE, Feinberg AP (1993) Relaxation of imprinted genes in human cancer. Nature 362: 747–749 8385745 10.1038/362747a0

[bib31] Ries LA (1999) Cancer Incidence And Survival Among Children And Adolescents: United States SEER Program 1975-1995 Vol. 99-4649 National Cancer Institute: Bethesda

[bib32] Ruymann FB, Maddux HR, Ragab A, Soule EH, Palmer N, Beltangady M, Gehan EA, Newton Jr WA (1988) Congenital anomalies associated with rhabdomyosarcoma: an autopsy study of 115 cases. A report from the Intergroup Rhabdomyosarcoma Study Committee (representing the Children′s Cancer Study Group, the Pediatric Oncology Group, the United Kingdom Children′s Cancer Study Group, and the Pediatric Intergroup Statistical Center). Med Pediatr Oncol 16: 33–39 3277029 10.1002/mpo.2950160109

[bib33] Samuel DP, Tsokos M, DeBaun MR (1999) Hemihypertrophy and a poorly differentiated embryonal rhabdomyosarcoma of the pelvis. Med Pediatr Oncol 32: 38–43 9917751 10.1002/(sici)1096-911x(199901)32:1<38::aid-mpo8>3.0.co;2-h

[bib34] Schuz J, Kaletsch U, Meinert R, Kaatsch P, Michaelis J (2001) High-birth weight and other risk factors for Wilms tumour: results of a population-based case-control study. Eur J Pediatr 160: 333–338 11421411 10.1007/pl00008443

[bib35] Scrable H, Witte D, Shimada H, Seemayer T, Sheng WW, Soukup S, Koufos A, Houghton P, Lampkin B, Cavenee W (1989) Molecular differential pathology of rhabdomyosarcoma. Genes Chromosomes Cancer 1: 23–35 2487144 10.1002/gcc.2870010106

[bib36] Spector LG, Puumala SE, Carozza SE, Chow EJ, Fox EE, Horel S, Johnson KJ, McLaughlin CC, Reynolds P, Behren JV, Mueller BA (2009) Cancer risk among children with very low birth weights. Pediatrics 124: 96–104 19564288 10.1542/peds.2008-3069 PMC2704984

[bib37] Steliarova-Foucher E, Stiller C, Lacour B, Kaatsch P (2005) International classification of childhood cancer, third edition. Cancer 103: 1457–1467 15712273 10.1002/cncr.20910

[bib38] Tekin M, Dogu F, Tacyildiz N, Akar E, Ikinciogullari A, Ogur G, Yavuz G, Babacan E, Akar N (2002) 657del5 mutation in the NBS1 gene is associated with Nijmegen breakage syndrome in a Turkish family. Clin Genet 62: 84–88 12123493 10.1034/j.1399-0004.2002.620112.x

[bib39] Trahair T, Andrews L, Cohn RJ (2007) Recognition of Li Fraumeni syndrome at diagnosis of a locally advanced extremity rhabdomyosarcoma. Pediatr Blood Cancer 48: 345–348 16534790 10.1002/pbc.20795

[bib40] Valero De Bernabe J, Soriano T, Albaladejo R, Juarranz M, Calle ME, Martinez D, Dominguez-Rojas V (2004) Risk factors for low birth weight: a review. Eur J Obstet Gynecol Reprod Biol 116: 3–15 15294360 10.1016/j.ejogrb.2004.03.007

[bib41] Wachtel M, Runge T, Leuschner I, Stegmaier S, Koscielniak E, Treuner J, Odermatt B, Behnke S, Niggli FK, Schafer BW (2006) Subtype and prognostic classification of rhabdomyosarcoma by immunohistochemistry. J Clin Oncol 24: 816–822 16391296 10.1200/JCO.2005.03.4934

